# Health-related quality of life and depressive symptoms of patients with chronic diseases and the general population before and during the COVID-19 pandemic in Korea

**DOI:** 10.3389/fpsyg.2023.1117369

**Published:** 2023-02-09

**Authors:** Yeeun Park, Kyong Park

**Affiliations:** Department of Food and Nutrition, Yeungnam University, Gyeongsan, Gyeongbuk, Republic of Korea

**Keywords:** anxiety, COVID-19, chronic disease, depressive symptoms, Korea, quality of life

## Abstract

**Objective:**

The unprecedented coronavirus disease 2019 (COVID-19) outbreak has resulted in a global crisis that negatively impacted physical well-being and mental health. Our goal was to investigate the impact of the COVID-19 pandemic on health-related quality of life (HRQoL) and depressive symptoms in patients with chronic diseases and the general population in Korea.

**Methods:**

Data from 8341 patients with chronic diseases and 12,395 general population aged ≥20 years who participated in the Korea National Health and Nutrition Examination Survey (2017–2020) were analyzed. Patients with hypertension, dyslipidemia, diabetes, cerebrovascular disease (stroke), heart disease (myocardial infarction or angina pectoris), or cancer were classified as patients with chronic diseases. The general population was defined as those not suffering from corresponding chronic diseases. A modified EuroQol-5 Dimensions (EQ-5D), with three levels (0: extreme problems; 0.5: some problems; 1: no problems) for each dimension in EQ-5D, was used to assess HRQoL. To analyze depressive symptoms among patients with chronic diseases and the general population, we used the Patient Health Questionnaire-9 (PHQ-9) and defined a PHQ-9 score ≥ 10 as having a depressive symptom. Multivariate linear and logistic regression analyses were used to analyze HRQoL and depressive symptoms before and during the COVID-19 pandemic.

**Results:**

The HRQoL level was significantly lower in patients with chronic diseases compared to the general population on all dimensions both before and during the COVID-19 pandemic (all value of *p* < 0.05). Patients with chronic diseases had significantly lower HRQoL levels associated with the anxiety/depression dimension during the COVID-19 pandemic than in the pre-pandemic period (0.940 ± 0.002 vs. 0.929 ± 0.004, value of *p* = 0.041). In addition, patients with chronic diseases were more likely to report depressive symptoms during the COVID-19 pandemic than in the pre-pandemic period (Odds ratio (OR): 1.755, 95% confidence interval (CI): 1.209–2.546, value of *p* = 0.003). However, this association was not observed in the general population (OR: 1.275, 95% CI: 0.933–1.742, value of *p* = 0.13).

**Conclusion:**

The COVID-19 pandemic affected the HRQoL and psychological health in patients with chronic diseases with higher anxiety/depression during the pandemic than in the pre-pandemic period. These results suggest that it is urgent to establish continuous management guidelines, including psychosocial management for high-risk groups, and to improve the existing healthcare system.

## 1. Introduction

The emergence of the coronavirus disease 2019 (COVID-19) pandemic has resulted in a worldwide public health crisis (World Health Organization, 2020a,c), with sudden alterations to daily routines stemming from restrictions on social interactions, physical distancing measures, and isolation protocols potentially leading to psychological harm, including feelings of loneliness, fear, anxiety, and depression (Brooks et al., 2020; Lee, 2020; Luo M. et al., 2020; Talevi et al., 2020; Dorri et al., 2021). Studies have shown that COVID-19 had a negative effect on the quality of life (QoL) and psychological health, such as anxiety and depression (Brooks et al., 2020; Luo M. et al., 2020; Talevi et al., 2020; Dorri et al., 2021), as reflected by the new term “Corona Blues” (combining COVID-19 and blues, meaning depression) (Lee, 2020). A study in China with 1593 participants aged ≥18 years reported that those affected by quarantine had a higher prevalence of anxiety (12.9% vs. 6.7%) and depression (22.4% vs. 11.9%) than those unaffected by quarantine during the COVID-19 pandemic (Lei et al., 2020). Moreover, according to a study from the US including 1441 adults aged ≥18 years, the prevalence of depressive symptoms during the pandemic increased threefold to 27.8% compared with the prevalence obtained by the 2017–2018 National Health and Nutrition Examination Survey that included data on depressive symptoms of 5065 participants (Ettman et al., 2020). In South Korea, the Korean Society of Traumatic Stress Studies conducted a COVID-19 National Mental Health Survey, which revealed that the prevalence of suicidal ideation in June 2022 was 12.7%, which remained elevated in comparison to the 9.7% recorded in March 2020, during the initial phase of the COVID-19 outbreak (Ministry of Health and Welfare, 2022). Additionally, a one-year analysis of anxiety and depression levels following the initial COVID-19 outbreak reported a 55.8% prevalence, representing an 8.3% increase from 2020 (Lee and Kim, 2021). Consequently, in light of these findings, the World Health Organization (WHO) has emphasized the necessity for providing psychosocial support to address the mental burden associated with the COVID-19 pandemic (World Health Organization, 2020b).

The COVID-19 outbreak has also overloaded the healthcare system (e.g., due to limited manpower due to infection among healthcare workers, and shortages of hospital beds, equipment, or medicine) (Jazieh et al., 2020; Ennab and Ibdah, 2021; Romano et al., 2021; Shin et al., 2021). Indeed, some hospitals were forced to suspend regular healthcare services temporarily to respond to COVID-19 (Jazieh et al., 2020; Breast Screening Working Group (WG2) of the COVID-19 and Cancer Global Modelling Consortium et al., 2021), and patients have avoided hospital visits to limit the risk of COVID-19 infection (Cheng et al., 2021; Hammad et al., 2021; Kim et al., 2021). The above circumstances contributed to delays in diagnosis, management, or treatment of disease to some extent (de Joode et al., 2020; Jazieh et al., 2020; Breast Screening Working Group (WG2) of the Covid-19 and Cancer Global Modelling Consortium et al., 2021). Patients with chronic diseases such as hypertension, dyslipidemia, diabetes, cerebrovascular disease, heart disease, or cancer–collectively referred to as patients with underlying medical conditions, who have a higher risk of developing serious illness due to COVID-19 (Aggarwal et al., 2020; Dai et al., 2020; Liang et al., 2020; Zhou et al., 2020; Centers for Disease Control and Prevention, 2022c) might experience psychological burden (de Joode et al., 2020; Kayikcioglu et al., 2020; Wang Y. et al., 2020; Kim and Kim, 2022a,c; Ozkan et al., 2022). For example, a retrospective study in the UK analyzed the impact of COVID-19 on patient-reported health outcomes at a 30-day follow-up after hospitalization for acute stroke (Ozkan et al., 2022). Patients hospitalized during the pandemic (n = 95) had exacerbation of anxiety, depression, fatigue, pain, and sleep disturbance and reduced social participation and physical function compared with those hospitalized before the COVID-19 pandemic (*n* = 106) (Ozkan et al., 2022). In addition, a study conducted in Turkey assessing the impact of the COVID-19 pandemic on 169 patients with a previous history of premature myocardial infarction indicated that about two-thirds of the study patients reported an increased anxiety level (Kayikcioglu et al., 2020).

The QoL is a multidimensional concept that includes the subjective health status that an individual perceives, and health-related quality of life (HRQoL) can be useful for setting health goals and measuring prognosis among clinical patients (Yun et al., 2004; Nam et al., 2007; Centers for Disease Control and Prevention, 2018). Depression is one of the most seriously considered diseases worldwide, and it is also a risk factor for chronic diseases (Keck, 2010). In addition, the Patient Health Questionnaire-9 (PHQ-9) is widely used as a screening tool to detect depression and depressive symptoms (Park et al., 2010). Given the significance of evaluating the impact of the COVID-19 pandemic on HRQoL and depressive symptoms, particularly in individuals with chronic conditions, it is crucial to conduct assessments in order to establish systematic strategies for preserving and promoting HRQoL and mental well-being for both the population with chronic diseases and the general population. Several studies have reported the mental health of patients with chronic diseases since the COVID-19 outbreak using data from 2020. However, some analyses were without data on mental health status before the pandemic (Ahn et al., 2020; Kim et al., 2021; Kim and Kim, 2022c), while others focused on a specific sex group or on patients with a specific type of cancer (Park et al., 2022; Kim and Kim, 2022b).

In this study, our objective was to comparatively examine the impact of the COVID-19 pandemic on the HRQoL and depressive symptoms in patients with chronic diseases and the general population in South Korea, by utilizing the Korea National Health and Nutrition Examination Survey (KNHANES) 2017–2020, an annual nationally representative survey. We aimed to achieve this by comparing data collected pre-pandemic and during the pandemic period.

## 2. Methods

### 2.1. Study population

The KNHANES is a large-scale cross-sectional survey conducted by extracting representative samples of the Korean population to identify health status and behaviors, the prevalence of chronic diseases, and food and nutrient intake and to use the findings for creating health policies (Korea Disease Control and Prevention Agency, 2020c). The survey was conducted in 3-year cycles from the first wave in 1998 to the third wave in 2005. Starting from the fourth wave in 2007, the survey was conducted annually after adopting the rolling sample survey format. Data from the eighth wave second year (2020) was recently released. The seventh (2016–2018) and eighth waves (2019–2020) of the KNHANES utilized population and housing census data as the foundation for their sampling frames. In particular, the seventh wave sought to improve the population inclusion rate by incorporating data on Officially Announced Prices for Apartment Housing. The target population for the KNHANES is comprised of individuals aged one and above who reside in Korea, and a representative sample is drawn through a multi-stage clustered probability design. Specifically, the primary sampling unit is the enumeration district, while the secondary sampling unit is the household. The KNHANES largely consists of a health questionnaire survey, health examination, and nutritional survey. An explanation of the survey design and analytical methods have been described in detail in previous studies (Kweon et al., 2014).

The present study analyzed the KNHANES data from 2017 to 2020 to compare data from before and during the COVID-19 pandemic. The pre-pandemic period was defined as 2017–2019 and the COVID-19 pandemic period was defined as 2020.

The study population included two groups: (1) patients with chronic diseases and (2) the general population. The group of patients with chronic diseases included those with hypertension, dyslipidemia, diabetes, cerebrovascular disease (stroke), heart disease (myocardial infarction or angina pectoris), or cancer. These patients were classified as “patients with chronic diseases” if they met the following criteria: (1) diagnosed by a physician, (2) currently suffering from the disease, (3) have been treated for the disease, or (4) taking medicines related to the disease. Cancer was defined as having at least one of the following types: gastric, liver, colorectal, breast, cervical, lung, thyroid, and other cancers. The general population was defined as those who did not have any of the corresponding chronic diseases.

In the KNHANES 2017–2020 data, 31,588 participants were initially included in the study. However, the following respondents were excluded from the final analysis: (1) those under the age of 20 (*n* = 6092) and (2) respondents with missing statistical weight values (*n* = 4760). As a result, a total of 20,736 individuals were included in the final analysis, with 8341 classified as patients with chronic diseases and 12,395 classified as part of the general population.

Research data from all participants in the KNHANES were used with approval from the Institutional Review Board (IRB) of the Korea Disease Control and Prevention Agency (KDCA) (approval number: 2018-01-03-P-A, 2018-01-03-C-A, 2018-01-03-2C-A). The KNHANES 2017 was conducted without review, according to the IRB of the KDCA.

### 2.2. Assessment of demographic and lifestyle information

Health behavior information, such as alcohol consumption and smoking status, was collected using a self-reporting format, whereas a trained staff collected information on sex, age, education level, physical activity, QoL, PHQ-9, and morbidities through an interview (Korea Disease Control and Prevention Agency, 2020c). The average monthly equalized household income was calculated by considering the total age-and sex-specific annual income of the household members (Korea Disease Control and Prevention Agency, 2020c) and was categorized as low, mid-low, mid-high, and high. Education level was reclassified into two categories: less than high school education and high school educated or higher. Alcohol consumption was calculated by multiplying the daily frequency of alcohol consumption by the amount of alcohol consumed at one time. For smoking status, the respondents were classified according to whether they smoke every day or occasionally (current smokers), used to smoke but not anymore (former smokers), and never smoked (non-smokers). Anthropometric data were collected by trained staff. Body mass index (BMI) was calculated as the weight (kg) divided by height squared (*m*^2^). For physical activity, the metabolic equivalent task-hours per week (METs-h/week) were calculated, and weight was assigned according to the intensity of each exercise (Ainsworth et al., 1993).

### 2.3. HRQoL measuring tool

To analyze HRQoL among patients with chronic diseases and the general population, the present study used the EuroQol-5 Dimensions (EQ-5D), a tool used in the KNHANES to measure HRQoL. The EQ-5D is comprised of five dimensions: mobility, self-care, usual activities, pain/discomfort, and anxiety/depression (Rabin and de Charro, 2001). The validity of EQ-5D in Korea has been evaluated by studies on patients with rheumatic diseases and stroke (Kim et al., 2005; Jo and Bae, 2009). The present study reclassified the existing questions for each dimension in EQ-5D to three levels: 0, extreme problems; 0.5, some problems; 1, no problems. The EQ-5D index, which combines each dimension from EQ-5D, was calculated through the EQ-5D Korean Valuation Study Using Time Trade Off Method (Nam et al., 2007). Further detailed information on this weight was described previously (Nam et al., 2007).

### 2.4. Depressive symptom screening tool

To analyze depressive symptoms among patients with chronic diseases and the general population, the present study used the PHQ-9, screened biennially in the KNHANES. For this analysis, we used data from KNHANES 2018 and 2020, where the PHQ-9 was available for the analysis (Korea Disease Control and Prevention Agency, 2020c). Validated PHQ-9 (Park et al., 2010) consists of nine questions, and each question has a score of 0 (not at all) to 3 (nearly every day). The distribution of scores is from 0 to 27, and the higher the total score, the more severe the symptoms related to depression. In this study, we defined a PHQ-9 score ≥ 10 as having a depressive symptom (Kroenke et al., 2001).

### 2.5. Statistical analysis

Based on the complex sampling design of the KNHANES, analyses in the present study were performed by considering all stratification variables, cluster variables, and weights. Categorical variables are presented as frequency and percentage, and continuous variables are presented as mean ± standard error. A multivariate general linear model was conducted to investigate the mean differences in EQ-5D between patients with chronic diseases and the general population across the pre-and pandemic periods. A multivariate logistic regression analysis was performed to examine the prevalence of depressive symptoms in patients with chronic diseases and the general population, in relation to the pre-and pandemic periods of the COVID-19 pandemic; the estimates are presented as the odds ratio (OR) and 95% confidence interval (CI). Potential confounding variables were considered through a preliminary analysis and by reviewing previous studies (Kim and Kim, 2018; Choi et al., 2020; Ettman et al., 2020; Cerezo and Vicario, 2021; Jeppesen et al., 2021; Adzrago et al., 2022; Myers et al., 2022), and sex, age, household income, education level, alcohol consumption, smoking status, BMI, and physical activity were included as covariates. Significant effect modification was not observed in the association between the survey year (the pre-and pandemic periods of the COVID-19 pandemic) and HRQoL/depressive symptoms. All statistical analyses in the present study were performed using SAS 9.4 version (Statistical Analysis System; SAS Institute Inc., Cary, NC, United States), with the statistical significance level set to α = 0.05.

## 3. Results

### 3.1. Characteristics of the participants across the pre-and pandemic periods of the COVID-19

In the pre-pandemic period (2017–2019), the number of patients with hypertension, dyslipidemia, diabetes, stroke, myocardial infarction/angina, and cancer among those with chronic diseases were 4174, 3202, 1603, 411, 491, and 900, respectively. During the pandemic period (2020), the number of patients with hypertension, dyslipidemia, diabetes, stroke, myocardial infarction/angina, and cancer among those with chronic diseases were 1226, 1062, 553, 103, 144, and 253, respectively.

Table 1 presents the analysis of demographic characteristics and lifestyle factors among patients with chronic diseases and the general population, in relation to the pre-and pandemic periods of the COVID-19 pandemic. In the sample of 8341 patients with chronic diseases, 6404 participants were surveyed in the pre-pandemic period (2017–2019) and 1937 were surveyed during the pandemic period (2020). Similarly, among the 12,395 members of the general population, 9780 were surveyed in the pre-pandemic period (2017–2019) and 2615 were surveyed during the pandemic period (2020).

**Table 1:** General characteristics of patients with chronic diseases and the general population across the pre-and the pandemic periods of the COVID-19, KNHANES 2017–2020.

	Patients with chronic diseases (*N* = 8,341)		General population (*N* = 12,395)	
	Pre (2017–2019)	Pandemic period (2020)	Pre (2017–2019)	Pandemic period (2020)
Sex				
Men	2748 (42.9)	836 (43.2)	4142 (42.4)	1127 (43.1)
Women	3656 (57.1)	1101 (56.8)	5638 (57.6)	1488 (56.9)
*Age (years)*	64.401 ± 0.147	64.532 ± 0.267	44.900 ± 0.150	44.733 ± 0.304
Household income				
Low	2086 (32.7)	598 (31.0)	1167 (12.0)	298 (11.4)
Mid-low	1678 (26.3)	487 (25.2)	2353 (24.1)	589 (22.6)
Mid-high	1364 (21.4)	426 (22.1)	2893 (29.7)	812 (31.2)
High	1256 (19.6)	421 (21.7)	3340 (34.2)	907 (34.8)
Education level				
Lower than high school education	3287 (53.8)	846 (49.3)	1449 (15.6)	310 (12.7)
High school educated or higher	2820 (46.2)	869 (50.7)	7839 (84.4)	2139 (87.3)
*Alcohol consumption* ^a^	0.700 ± 0.019	0.637 ± 0.033	0.804 ± 0.015	0.757 ± 0.028
Smoking status				
Non-smokers	3774 (59.5)	1153 (60.2)	6014 (62.1)	1616 (62.3)
Former smokers	1717 (27.1)	486 (25.4)	1882 (19.4)	530 (20.4)
Current smokers	851 (13.4)	278 (14.4)	1791 (18.5)	450 (17.3)
*Body mass index (kg/m*^*2*^)	24.669 ± 0.043	24.841 ± 0.080	23.455 ± 0.036	23.696 ± 0.075
*Physical activity (METs-h/week)*	15.377 ± 0.276	15.831 ± 0.464	16.770 ± 0.225	17.118 ± 0.397

COVID-19, Coronavirus Disease 2019; KNHANES, Korea National Health and Nutrition Examination Survey; METs-h/week, Metabolic equivalent task-hours per week. Values are presented as *n* (%) or mean ± standard error. ^a^The unit of alcohol consumption is the number of standard drinks per day.

The average age of patients with chronic diseases was approximately 64.40–64.53 years, while the average age of the general population was approximately 44.73–44.90 years. The proportion of individuals with higher education (high school graduation or higher) was approximately 50% among patients with chronic diseases, compared to 84–87% in the general population. The BMI of patients with chronic diseases and the general population were 24.67–24.84 kg/m^2^ and 23.46–23.70 kg/m^2^, respectively. The physical activity levels of patients with chronic diseases and the general population were 15.38–15.83 and 16.77–17.12 METs-h/week, respectively. Other characteristics, such as sex, alcohol consumption, and smoking status, were fairly similar between the two groups of patients with chronic diseases and the general population.

### 3.2. Comparison of HRQoL between patients with chronic disease and the general population across the pre-and pandemic periods of the COVID-19

A multivariate linear regression analysis was conducted to calculate the adjusted mean levels of EQ-5D as the outcome variable, and to compare these levels between patients with chronic disease and the general population (using two different population groups as the predictor variable) separately for the pre-pandemic period and the COVID-19 pandemic period (Table 2). After adjusting for all potential confounding factors, the results indicate that the individual components of EQ-5D were significantly lower in patients with chronic diseases compared to the general population on all dimensions, including mobility, self-care, usual activities, pain/discomfort, and anxiety/depression, both before and during the COVID-19 pandemic (all *p* < 0.05). Additionally, the EQ-5D index was also significantly lower in patients with chronic diseases compared to the general population, both before and during the COVID-19 pandemic (*p* < 0.001).

**Table 2:** Adjusted mean comparison of EQ-5D between patients with chronic diseases and the general population during the pre-and pandemic periods of the COVID-19.

EQ-5D^a^	Pre-pandemic (2017–2019) (*N* = 16,184)			Pandemic period (2020) (*N* = 4,552)		
	Patients with chronic diseases (*N* = 6,404)	General population (*N* = 9,780)	*p*-value	Patients with chronic diseases (*N* = 1,937)	General population (*N* = 2,615)	*p*-value
Mobility	0.896 ± 0.003	0.916 ± 0.003	<0.001	0.905 ± 0.006	0.920 ± 0.004	0.007
Self-care	0.972 ± 0.002	0.979 ± 0.001	<0.001	0.972 ± 0.004	0.982 ± 0.003	<0.001
Usual activities	0.939 ± 0.003	0.957 ± 0.002	<0.001	0.946 ± 0.005	0.963 ± 0.003	<0.001
Pain/Discomfort	0.844 ± 0.005	0.861 ± 0.004	0.002	0.849 ± 0.009	0.883 ± 0.007	0.002
Anxiety/Depression	0.936 ± 0.003	0.948 ± 0.002	<0.001	0.928 ± 0.005	0.947 ± 0.005	0.004
EQ-5D index	0.928 ± 0.002	0.940 ± 0.002	<0.001	0.931 ± 0.004	0.946 ± 0.003	<0.001

EQ-5D, EuroQol-5 Dimensions; COVID-19, Coronavirus Disease 2019. ^a^Each dimension was reclassified into three levels: no problems (1), some problems (0.5), and extreme problems (0). Values are mean levels of EQ-5D ± standard error, adjusting for sex (men and women), age (continuous), household income (low, mid-low, mid-high, and high), education level (less than high school education and high school educated or higher), alcohol consumption (continuous), smoking status (current smokers, former smokers, and non-smokers), body mass index (continuous), and physical activity (continuous).

### 3.3. Comparison of HRQoL in patients with chronic diseases across the pre-and pandemic periods of the COVID-19

A multivariate linear regression analysis was employed to determine the adjusted mean levels of EQ-5D as the outcome variable, and to compare these levels between the pre-pandemic period and the COVID-19 pandemic period (using different survey years as the predictor variable) in patients with chronic disease (Table 3). After adjusting for all confounding factors, the results indicate that the HRQoL level in the anxiety/depression dimension was significantly lower during the COVID-19 pandemic in comparison to the pre-pandemic period (mean ± standard error: 0.940 ± 0.002 vs. 0.929 ± 0.004, *p* = 0.041). However, there were no significant differences in HRQoL levels in the other dimensions (all *p* > 0.05).

**Table 3:** Adjusted mean values of EQ-5D before and during the COVID-19 pandemic in patients with chronic diseases.

EQ-5D^a^	Patients with chronic diseases (*N* = 8,341)		
	Before COVID-19 (2017–2019)	During COVID-19 (2020)	*p*-value
Mobility	0.864 ± 0.004	0.873 ± 0.006	0.12
Self-care	0.962 ± 0.002	0.962 ± 0.004	0.81
Usual activities	0.928 ± 0.005	0.934 ± 0.006	0.23
Pain/Discomfort	0.825 ± 0.005	0.834 ± 0.008	0.28
Anxiety/Depression	0.940 ± 0.002	0.929 ± 0.004	0.041
EQ-5D index	0.915 ± 0.003	0.918 ± 0.004	0.32

EQ-5D, EuroQol-5 Dimensions; COVID-19, Coronavirus Disease 2019. Values are presented as mean ± standard error. ^a^Each dimension was reclassified into three levels: no problems (1), some problems (0.5), and extreme problems (0). Values are adjusted for sex (men and women), age (continuous), household income (low, mid-low, mid-high, and high), education level (less than high school education and high school educated or higher), alcohol consumption (continuous), smoking status (current smokers, former smokers, and non-smokers), body mass index (continuous), and physical activity (continuous).

### 3.4. Comparison of depressive symptoms in patients with chronic diseases and the general population across the pre-and pandemic periods of the COVID-19

A multivariate logistic regression analysis was conducted to calculate odds ratios and confidence intervals for depressive symptoms (based on a PHQ-9 score of ≥ 10) as the outcome variable. The analysis compared the odds of depressive symptoms between the pre-pandemic period (2018) and the COVID-19 pandemic period (2020) separately for patients with chronic diseases (*n* = 4041) and the general population (*n* = 5921) by using different survey years as the predictor variable. The results of this analysis are presented in Table 4. The results indicate that among patients with chronic diseases, the prevalence of depressive symptoms was significantly higher during the COVID-19 pandemic in comparison to the pre-pandemic period, as evidenced by all statistical models (Model 3 OR: 1.755, 95% CI: 1.209–2.546, *p* = 0.003). On the other hand, among the general population, the prevalence of depressive symptoms did not differ between the pre-and pandemic periods of the COVID-19 pandemic, as supported by all statistical models (Model 3 OR: 1.275, 95% CI: 0.933–1.742, *p* = 0.13).

**Table 4:** Change in the prevalence of depressive symptoms between the pre-pandemic and during pandemic periods in patients with chronic diseases and the general population.

PHQ-9 score ≥ 10^a^	Before COVID-19 (2018)	During COVID-19 (2020)	*p*-value
Patients with chronic diseases (*N* = 4,041)			
Model 1	Ref	1.632 (1.141–2.336)	0.008
Model 2	Ref	1.615 (1.135–2.299)	0.008
Model 3	Ref	1.755 (1.209–2.546)	0.003
General population (*N* = 5,921)			
Model 1	Ref	1.222 (0.893–1.674)	0.21
Model 2	Ref	1.224 (0.895–1.673)	0.20
Model 3	Ref	1.275 (0.933–1.742)	0.13

PHQ, Patient Health Questionnaire; COVID-19, Coronavirus Disease 2019. ^a^PHQ-9 score ≥ 10 was considered to have depressive symptoms. Values are presented as odds ratios (95% confidence intervals). Model 1: unadjusted. Model 2: adjusted for sex (men and women) and age (continuous). Model 3: additionally adjusted for household income (low, mid-low, mid-high, and high), education level (less than high school education and high school educated or higher), alcohol consumption (continuous), smoking status (current smokers, former smokers, and non-smokers), body mass index (continuous), and physical activity (continuous).

## 4. Discussion

The current study examined data from the KNHANES 2017–2020 to compare the HRQoL levels and depressive symptoms among patients with chronic diseases and the general population before and during the COVID-19 pandemic. The analysis revealed that the HRQoL level was significantly lower in patients with chronic diseases compared to the general population both before and during the COVID-19 pandemic. Furthermore, patients with chronic diseases had significantly lower HRQoL levels in the anxiety/depression dimension during the COVID-19 pandemic in comparison to the pre-pandemic period. Additionally, patients with chronic diseases were more likely to experience depressive symptoms during the COVID-19 pandemic. In contrast, the general population did not exhibit a significant difference in the prevalence of depressive symptoms before and during the COVID-19 pandemic.

Previous research has reported that individuals with chronic diseases experience a negative impact on their QoL due to the prolonged nature of treatment and management, and that chronic diseases and mental health are interrelated (Chapman et al., 2005; Calvert et al., 2012; Megari, 2013; Ryoung and Byung-Deog, 2014; Cho, 2021; Centers for Disease Control and Prevention, 2022a). Consistent with these findings, this study revealed that compared to the general population, patients with chronic diseases exhibited lower HRQoL levels in all dimensions, both before and during the COVID-19 pandemic. Furthermore, the COVID-19 pandemic may exacerbate the risk for depression among individuals with chronic illnesses (Chen et al., 2020; Özdin and Bayrak Özdin, 2020; Luo Y. et al., 2020; Wang C. et al., 2020). According to a meta-analysis to assess the levels and prevalence of anxiety, distress, and stress in patients with diabetes during the COVID-19 pandemic, the prevalence of anxiety in type 2 diabetes patients was 20 and 36% in diabetes distress (García-Lara et al., 2022), which were higher than those in the pre-pandemic period [18% (Chaturvedi et al., 2019) and 29.4% (Huynh et al., 2021), respectively]. Similarly, a study in China on 658 patients with breast cancer found that severe anxiety and depression during the COVID-19 pandemic were 8.9 and 9.3%, respectively. This was higher than the results of previous studies of breast cancer patients in China before the COVID-19 pandemic (severe anxiety = 3.5%, moderate to severe depression = 3.5%) (Juanjuan et al., 2020; Lan et al., 2020).

Patients with chronic diseases with weakened immune systems have been identified as a high-risk group for severe COVID-19 infection and its related complications (Centers for Disease Control and Prevention, 2022c), as reported in studies on cancer patients (Dai et al., 2020; Liang et al., 2020; Ofori-Asenso et al., 2020). Therefore, individuals with chronic illnesses may experience increased psychological anxiety due to the fear of COVID-19 exposure. Additionally, disruptions to medical care caused by the outbreak of COVID-19, such as delays or suspensions in testing and treatment (de Joode et al., 2020; Jazieh et al., 2020; Breast Screening Working Group (WG2) of the Covid-19 and Cancer Global Modelling Consortium et al., 2021), are likely to have added a significant mental burden on patients with chronic diseases who require continuous treatment (Guven et al., 2020; Singhai et al., 2020; Kim et al., 2021). According to a study that analyzed the impact of the COVID-19 lockdown on patients with chronic diseases, it was reported that 42% of 181 patients missed regular testing (Saqib et al., 2020). Indeed, a study in China on 141 cancer outpatients reported that 41.8% experienced a delay in treatment, 60.3% feared visiting a hospital, and 85.1% were concerned about treatment delay (Liu et al., 2021). Similarly, a study on 154 patients with breast cancer reported that 18.8% of the patients experienced changes in treatment due to COVID-19, and those who experienced a treatment plan change had higher levels of depression than those who did not (Kim and Kim, 2022b).

Meanwhile, the average age of the chronic disease patients in this study were 64 years old, including mainly the elderly. Elderly individuals have a higher likelihood of developing chronic diseases (National Cancer Information Center, 2019; Korea Disease Control and Prevention Agency, 2020b, 2021; Statistics Korea, 2021). The elderly population, like patients with pre-existing conditions, is more vulnerable to COVID-19, and mortality rates due to COVID-19 are known to be higher than those reported for younger age groups (Wang L. et al., 2020; Centers for Disease Control and Prevention, 2022b,c). Indeed, according to a previous study among the elderly, the prevalence of clinically significant depressive symptoms among older adults increased to 19.8% during the COVID-19 pandemic, as compared to 7.2% before the pandemic onset (Briggs et al., 2021). Moreover, a deeper sense of isolation due to the temporary closure of senior welfare facilities and restrictions on social activities and social exchange since the COVID-19 pandemic could increase the emotional burden on the elderly (Shin et al., 2020). It is also believed that restrictions on visitors through strict control measures in nursing homes during the COVID-19 pandemic could negatively affect the mental health of the elderly in need of care (Chee, 2020).

Despite these interesting findings, the present study had some limitations. Firstly, while potential confounding factors were adjusted through the review of previous studies and a preliminary analysis, some unmeasured or unknown residual confounding factors (e.g., cancer stage, treatment type, treatment stage, and disease severity) that may affect the HRQoL and depressive symptoms of patients with chronic diseases may still exist. Secondly, this study analyzed the period after the COVID-19 pandemic outbreak by limiting it to 2020. There are regional differences in the timing of the COVID-19 epidemic, and HRQoL and the mental health status of patients with chronic diseases and the general population may change according to the different stages of the COVID-19 pandemic. Thus, it is necessary to investigate the long-term effects of COVID-19 on patients with chronic diseases and the general population. Thirdly, the present study was a cross-sectional study, and thus, the causal relationship between the HRQoL and depressive symptoms and COVID-19 could not be identified. Finally, in 2020, the number of participants decreased by about 750 compared with that in the last year due to the COVID-19 pandemic, and information about the HRQoL and depressive symptoms was collected through surveys. Thus, non-differential misclassification errors might have occurred due to these limitations. However, the KNHANES provides clear guidelines for health surveys (Korea Disease Control and Prevention Agency, 2020a), and all surveys were conducted in the same way as in 2017–2019 with the aid of trained staff to minimize the possibility of errors. Despite these limitations, the present study used highly reliable national sample data to analyze the HRQoL and depressive symptoms of Korean patients with chronic diseases and the general population before and during the COVID-19 pandemic.

Given the prolonged nature of the current pandemic, it is crucial to develop healthcare system guidelines for high-risk groups for infectious diseases. Our findings underscore the importance of enhancing the healthcare service environment to provide adequate psychosocial support during the COVID-19 pandemic, as well as fortifying the healthcare system in anticipation of potential future pandemics. Although we utilized the most up-to-date data available for analysis, we could only capture the initial phase of the COVID-19 outbreak. Therefore, follow-up studies are imperative to gain a more comprehensive understanding of the long-term negative effects of COVID-19 on patients with chronic illnesses, particularly with regards to the prolonged impact on HRQoL and mental health by incorporating data collected since 2020, as the pandemic is still ongoing. Finally, future studies should also investigate the effect of contracting COVID-19, by comparing patients with and without COVID-19 experience and examine the impact of changes in access and speed of care on patients’ mental health.

## Data availability statement

Publicly available datasets were analyzed in this study. This data can be found at: https://knhanes.kdca.go.kr/knhanes/main.do.

## Ethics statement

The studies involving human participants were reviewed and approved by Institutional Review Board of the Korea Disease Control and Prevention Agency. The patients/participants provided their written informed consent to participate in this study.

## Author contributions

YP contributed to the writing the original draft, formal analysis, visualization, and software. KP contributed to the conceptualization, supervision, project administration, resources, funding acquisition, validation, and discussion, along with editing the manuscript. All authors have read and agreed to the final version of the manuscript.

## Funding

This research was supported by the National Research Foundation of Korea grant funded by the Korea government (grant number: NRF-2021R1A2C1007869). The funding sponsor had no role in the study design; collection, analysis, and interpretation of data; writing of the report; and the decision to submit the article for publication.

## Conflict of interest

The authors declare that the research was conducted in the absence of any commercial or financial relationships that could be construed as a potential conflict of interest.

## Publisher’s note

All claims expressed in this article are solely those of the authors and do not necessarily represent those of their affiliated organizations, or those of the publisher, the editors and the reviewers. Any product that may be evaluated in this article, or claim that may be made by its manufacturer, is not guaranteed or endorsed by the publisher.
